# Prognostic and histologic significances of the expression profile of membrane tissue factor for aggressive endometrial carcinomas

**DOI:** 10.1093/oncolo/oyag053

**Published:** 2026-02-23

**Authors:** Kaoru Fujieda, Takeo Minaguchi, Kaori Ono, Takuya Kuboya, Asami Suto, Nan Qi, Hiroya Itagaki, Yuri Tenjimbayashi, Ayumi Shikama, Azusa Akiyama, Sari Nakao, Yusuke Kobayashi, Toyomi Satoh

**Affiliations:** Department of Obstetrics and Gynecology, Institute of Medicine, University of Tsukuba, Tsukuba, Ibaraki 305-8575, Japan; Department of Obstetrics and Gynecology, Institute of Medicine, University of Tsukuba, Tsukuba, Ibaraki 305-8575, Japan; Department of Obstetrics and Gynecology, Institute of Medicine, University of Tsukuba, Tsukuba, Ibaraki 305-8575, Japan; Department of Obstetrics and Gynecology, Institute of Medicine, University of Tsukuba, Tsukuba, Ibaraki 305-8575, Japan; Department of Obstetrics and Gynecology, Institute of Medicine, University of Tsukuba, Tsukuba, Ibaraki 305-8575, Japan; Doctoral Program in Obstetrics and Gynecology, Graduate School of Comprehensive Human Sciences, University of Tsukuba, Tsukuba, Ibaraki 305- 8575, Japan; Department of Obstetrics and Gynecology, Institute of Medicine, University of Tsukuba, Tsukuba, Ibaraki 305-8575, Japan; Department of Obstetrics and Gynecology, Institute of Medicine, University of Tsukuba, Tsukuba, Ibaraki 305-8575, Japan; Department of Obstetrics and Gynecology, Institute of Medicine, University of Tsukuba, Tsukuba, Ibaraki 305-8575, Japan; Department of Obstetrics and Gynecology, Institute of Medicine, University of Tsukuba, Tsukuba, Ibaraki 305-8575, Japan; Department of Obstetrics and Gynecology, Institute of Medicine, University of Tsukuba, Tsukuba, Ibaraki 305-8575, Japan; Department of Obstetrics and Gynecology, Institute of Medicine, University of Tsukuba, Tsukuba, Ibaraki 305-8575, Japan; Department of Obstetrics and Gynecology, Institute of Medicine, University of Tsukuba, Tsukuba, Ibaraki 305-8575, Japan

**Keywords:** tissue factor, endometrial carcinoma, aggressive histology, overall survival

## Abstract

**Background:**

Tissue factor (TF) is involved in tumor-induced coagulation cascade, which plays crucial roles in the tumor microenvironment, and is being clinically explored as a therapeutic target. However, the prognostic role of TF and the related proteins in endometrial cancer is yet to be clarified and was systematically investigated in this study.

**Materials and Methods:**

The expression profiles of membrane/cytoplasmic TF, nuclear/cytoplasmic phospho-TF (p-TF), PAR-1, PAR-2, VEGF as well as CD8, a marker for cytotoxic T cells, in tumors from 229 patients with endometrial carcinoma were immunohistochemically evaluated and correlated with clinicopathologic parameters and patient survival. Bioinformatics analyses were further conducted to strengthen the observations.

**Results:**

High membrane TF (mTF) expression correlated with worse overall survival (OS), and was found to be an independent prognostic factor for unfavorable OS by the univariate and multivariate analyses (*P* = .028 and .0087). High mTF expression correlated with aggressive histology, and remained independent for unfavorable OS even in the aggressive histological subset (*P* = .037 and .0064). Moreover, mTF expression inversely correlated with CD8^+^ tumor-infiltrating immune cell count, and TF expression positively correlated with the infiltration of Treg cells, known to suppress CD8^+^ T cells, by the The Cancer Genome Atlas (TCGA) data analysis (*P* = .037 and .0015), suggesting that the detrimental prognostic role of mTF involves immune evasion.

**Conclusions:**

Taken together, mTF serves as a potential biomarker for patient prognosis and therapeutic target for the aggressive histological type of tumor, providing significant rationales for incorporating TF-directed drugs into the novel strategy for refractory endometrial carcinomas.

Implications for Practice The incidence and mortality of endometrial cancer have been increasing over the past 2 decades in the United States, owing to rising rates of non-endometrioid histology which is clinically more aggressive than endometrioid subtype. Novel therapeutic strategy targeting aggressive histotypes is urgently required. Tisotumab vedotin, a TF-directed antibody-drug conjugate, has been recently approved by the FDA for advanced/recurrent cervical cancer. The expression profile and the prognostic significance of mTF for different histotypes by the present study suggest that tisotumab vedotin can be extendedly incorporated to the therapeutic strategy for endometrial carcinoma of aggressive histology, warranting verification by future clinical trials.

## Introduction

Endometrial cancer is the most common cancer of the female reproductive organ in the United States, with 69 120 new cases and 13 860 deaths estimated for 2025 (https://seer.cancer.gov/). The incidence and mortality rates have been increasing over the past 2 decades, owing to rising rates of non-endometrioid subtypes which are less common but more aggressive than endometrioid histology.^1^^,^^2^ Age-standardized 5-year survival by histologic subtype is reportedly 85.3%, 58.3%, 55.1%, and 38.0% for low-grade endometrioid, high-grade endometrioid, clear cell and serous histology, respectively.^3^ Novel therapeutic strategy targeting the aggressive histological subtypes of endometrial cancer is urgently required.

Tumor-induced coagulation pathway plays crucial roles in the tumor microenvironment (Figure S1—see online supplementary material for a color version of this figure).^4^ Tumor cells increase vessel permeability, angiogenic sprouting, and vasculogenic mimicry vessels, allowing extravasation of clotting factors and platelets from the blood stream into the stromal tissues. By activation and association with tissue factor (TF) which is expressed on the surface of tumor cells, factor VII (FVII) leaking from the blood vessels triggers the extrinsic coagulation pathway. TF-activated factor VII (FVIIa) complex activates factor X, which composes prothrombinase complex with factor V on the surface of activated platelets. Activation of thrombin follows with integrating the intrinsic coagulation cascade, resulting in platelets attachment and thrombus formation.^4^ In addition to thrombogenesis, TF is also involved in tumor growth, motility, adhesion and immune evasion, that is, escaping and inhibiting the immune system to detect and destruct tumor cells, through the following mechanisms.^5^^,^^6^ Activated thrombin in turn activates protease activated receptor-1 (PAR-1), which inhibits CD8a^+^ T cells infiltration. Via activated platelets and leukocytes/neutrophils, TF^+^ tumor cells adhere to the surface of vascular endothelial cells. Activated platelets then promote VEGF release, inducing neoangiogenesis, metastasis and dissemination. TF-FVIIa complex activates PAR-2 as well, which in turn activates PI3K and mitogen-activated protein kinase (MAPK) signaling cascades, leading to tumor growth.^6^ Activated PAR-2 also phosphorylates the cytoplasmic domain of TF, promoting cell migration and angiogenesis.^7^^,^^8^ VEGF-A binds to and activates VEGFR-2, transducing signals to PLCγ, PI3K/AKT, and p38-MAPK pathways, leading to tumor growth, invasion, and metastasis.^9^

The prognostic significance of TF expression has been investigated in malignancies including pancreatic, colon, gastric, esophageal, breast, prostate, kidney, and bladder cancers, indicating that TF is differentially expressed among histologic subtypes, and that TF overexpression generally correlates with poor prognosis.^10–12^ Moreover, p-TF alone as well as coexpression of p-TF and PAR-2 are reported to significantly correlate with shorter survival in breast cancer.^13^ As regards gynecological malignancies, TF expression increases thrombotic tendency in ovarian cancer, particularly clear cell carcinoma, which is characterized as chemoresistant histologic subtype.^14–16^ It is reported that high TF mRNA expression correlates with poor survival in ovarian cancer.^16^ In cervical cancer, TF is frequently overexpressed^17^ and significantly associated with advanced clinical stage, lymph node metastasis and distant metastasis.^18^ As for endometrial cancer, TF expression is reported to be associated with clear cell histology and with increased risk of venous thromboembolism (VTE).^19^ Tissue factor overexpression is reportedly frequent in endometrial serous carcinomas as well.^20^ Protease activated receptor-1 overexpression is confined to high-grade histologic subtypes,^21^ and PAR-2 expression correlates with advanced stages, high-grade histology and deep myometrial invasion.^22^ However, the precise prognostic and histologic significances of the expressions of TF and its related proteins in endometrial cancer is yet to be elucidated.

Tisotumab vedotin (TV) is a TF-directed antibody-drug conjugate (ADC), which binds to TF on target cells, is internalized, and releases monomethyl auristin E (MMAE). Monomethyl auristin E disrupts cellular microtubules and induces cell cycle arrest and apoptosis. Tisotumab vedotin-associated cytotoxicity is suggested to be enhanced by bystander effect, that is, antitumor activity against adjacent target-negative tumor cells, immunogenic cell death, and antibody-dependent toxicity and phagocytosis.^23^^,^^24^ In a phase 2 study on recurrent/metastatic cervical cancer (innovaTV 204/NCT03438396), TV monotherapy showed objective response rate of 24% with a tolerable safety profile.^25^ A randomized phase 3 trial of TV vs. chemotherapy in second- or third-line recurrent/metastatic cervical cancer (innovaTV 301/NCT04697628) showed significantly improved survival by TV.^26^ Based on this result, TV has been recently approved by the Food and Drug Administration (FDA) for the treatment of advanced/recurrent cervical cancer. However, to date no clinical trials have evaluated the efficacy and safety of TV for endometrial cancer. In order to explore the prognostic significance as well as to provide basic information on the expression of TF and the related proteins in this sort of tumor, we examined those expression profiles in endometrial carcinomas and correlated them with patient prognosis and clinicopathologic parameters including histologic subtypes. The current findings will offer significant implications for novel therapeutic option and useful information for formulating future clinical trials of TV in endometrial cancer.

##  Methods

### Patients and specimens

All patients diagnosed with clear cell carcinoma, serous carcinoma, or endometrioid carcinoma grade 3 (G3) of the endometrium who received primary surgery between January 2001 and December 2021 at the University of Tsukuba Hospital were identified through our database. A total of 100 patients with endometrioid carcinoma grade 1 (G1) were randomly selected from the database. A total of 229 patients (clear cell carcinoma, 23; serous carcinoma, 20; G3, 86; G1, 100) were included in the study (Figure S2—see online supplementary material for a color version of this figure). The study protocol was approved by the Clinical Research Ethics Committee of the University of Tsukuba Hospital (H26-118). All samples were obtained by the opt-out procedure in accordance with the national legislation.^27^ A median follow-up period excluding patients who died was 76 months (range, 4-268 months). Follow-up data were retrieved until 2023-9-8. Overall survival (OS) was defined as the interval between the start of primary treatment and death from any cause. Treatment-free interval (TFI) was defined as the interval between the end of primary adjuvant chemotherapy and the diagnosis of recurrence. Staging was conducted according to the criteria of the International Federation of Gynecology and Obstetrics (FIGO, 2008). We did not apply the latest criteria (FIGO, 2024), which incorporates histotype into staging, in order to separately assess histology and tumor extent. Treatment of patients was described previously.^28^  Table 1 summarizes the patient demographics.

**Table 1:** Patient demographics.

Characteristics	All histotypes (*n* = 229)	Aggressive histology (*n* = 129)	Endometrioid G1 (*n* = 100)	*P*-value
**Median age (range)**	61 (29-84)	62 ± 11	59 ± 11	0.062
**Median follow-up period (months)**	74.5 (4-264)	83 ± 45	85 ± 49	0.99
**Median BMI (range)**	23.9 (14.2-59.0)	24 ± 5	26 ± 7	0.023
**FIGO stage**				0.043
**I**	153 (67%)	78 (60%)	75 (75%)	
** IA**	111 (49%)	57 (44%)	54 (54%)	
** IB**	42 (18%)	21 (16%)	21 (21%)	
**II**	24 (10%)	13 (10%)	11 (11%)	
**III**	37 (16%)	26 (20%)	11 (11%)	
** IIIA**	14 (6%)	8 (6%)	6 (6%)	
** IIIC**	23 (10%)	18 (14%)	5 (5%)	
**IV**	15 (7%)	12 (9%)	3 (3%)	
** IVB**	15 (7%)	12 (9%)	3 (3%)	
**Myometrial invasion ≥1/2**	91 (40%)	55 (43%)	36 (36%)	0.34
**Histotype**				–
** Clear**	23 (10%)	23 (18%)	–	
** Serous**	20 (9%)	20 (16%)	–	
** Endometrioid G3**	86 (38%)	86 (67%)	–	
** Endometrioid G1**	100 (44%)	–	100 (100%)	
**Lymphovascular space invasion**	87 (38%)	63 (49%)	24 (24%)	0.00012
**Lymph node metastasis**	26 (11%)	20 (16%)	6 (6%)	0.0034
**Adjuvant chemotherapy**	70 (31%)	52 (40%)	18 (18%)	0.00029
** TC**	66 (30%)	49 (37%)	17 (17%)	
** CPT-N**	1 (0%)	1 (1%)	0 (0%)	
** CBDCA**	2 (1%)	2 (2%)	0 (0%)	
** CAP**	1 (0%)	0 (0%)	1 (1%)	
**Adjuvant radiotherapy**	62 (27%)	40 (31%)	22 (22%)	0.14
**VTE at the time of primary treatment or recurrence**	38 (17%)	26 (20%)	12 (12%)	0.11

Abbreviation: BMI, body mass index; CAP, cyclophosphamide, adriamycin and cisplatin; CBDCA, carboplatin; CPT-N, irinotecan and nedaplatin; FIGO, International Federation of Gynecology and Obstetricus; TC, paclitaxel and carboplatin; VTE, venous thromboembolism.

### Immunohistochemistry

Immunohistochemistry (IHC) procedures were described previously.^29^ Antibodies used were anti-TF (CD142) (mouse monoclonal, 1:50, BioMedica Diagnostics, Stamford, CT, USA), anti-p-TF (pSer 290) (rabbit polyclonal, 1:100, Sigma-Aldrich, St. Louis, MO, USA), anti-PAR-1 (ATAP2) (mouse monoclonal, 1:150, Santa Cruz, Dallas, TX, USA), anti-PAR-2 (SAM11) (mouse monoclonal, 1:100, Santa Cruz, Dallas, TX, USA), anti-VEGF (C-1) (mouse monoclonal, 1:50, Santa Cruz, Dallas, TX, USA), and anti-CD8 (clone C8/144B) (mouse monoclonal, 1:1, Nichirei Biosciences, Tokyo, Japan). Sections of umbilical cord (TF), brain (p-TF), kidney glomeruli (PAR-1) and tubules (PAR-1 and PAR-2), liver (VEGF) and lymph nodes (CD8) provided positive controls, and negative controls without addition of primary antibody showed low background staining.

### IHC scoring

**Figure 1:**
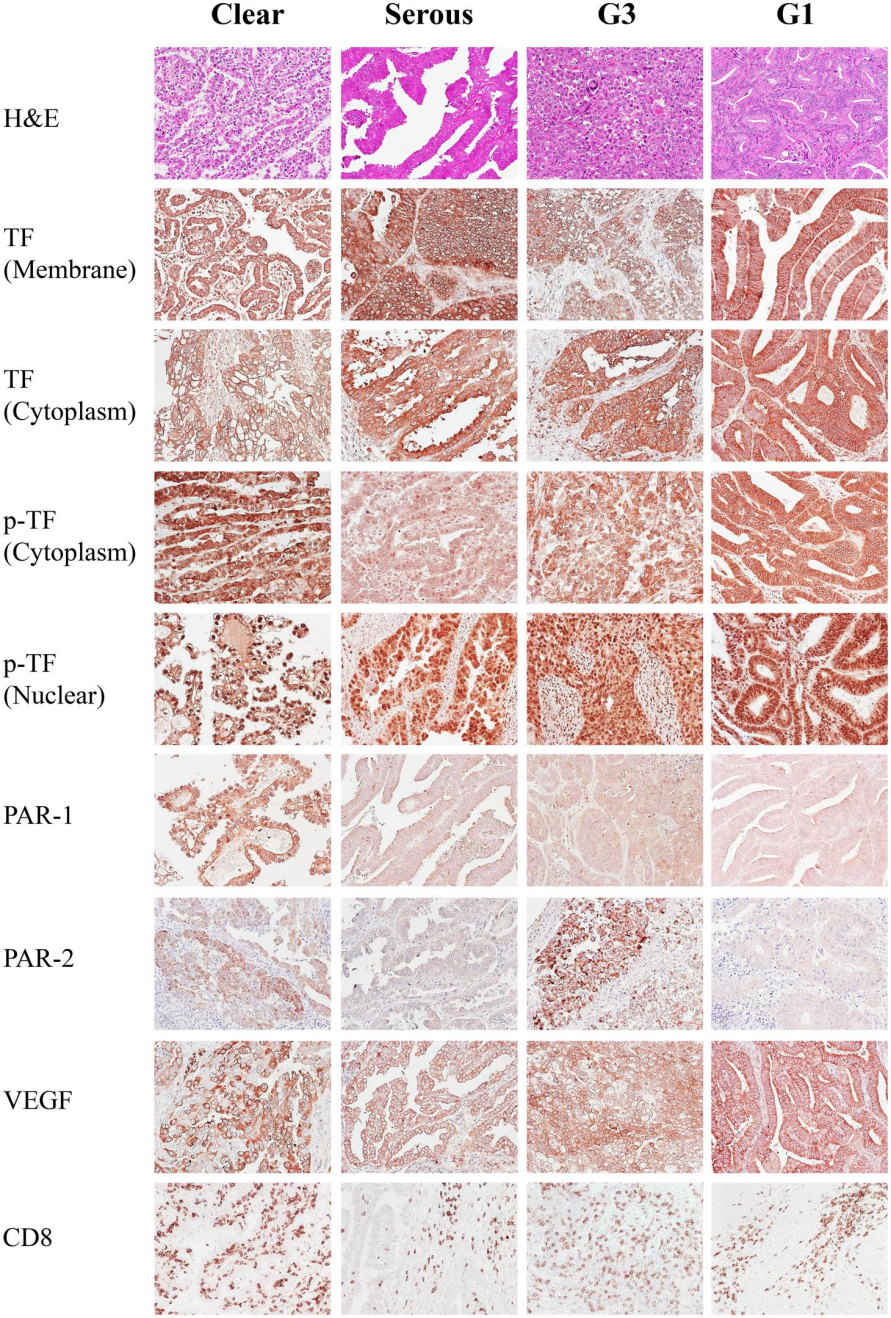
Representative images for immunohistochemistry of TF, the related proteins and CD8 in endometrial carcinomas (×200). For TF and the related proteins, cases with the degree of staining 2-3 are presented.

Semiquantitative immunoreactions were assigned by two investigators (K.F. and T.M.), and any discrepancies were resolved by conferring over a microscope. The IHC staining was scored by multiplying the percentages of positive tumor cells (0, no positive cell; 1, <10%; 2, 10%-50%; and 3, 50% < positive tumor cells) by the most prevalent degree of staining (0, no staining; 1, weak; 2, moderate; and 3, strong). The membrane and cytoplasmic stainings were separately evaluated for TF, and so were the nuclear and cytoplasmic stainings for p-TF. For CD8, we counted positive tumor-infiltrating immune cells (TICs). Three fields with most abundant tumor cells but without necrosis at magnification of ×200 were randomly selected from each slide, and the average IHC score or cell count was calculated. The representative images are displayed in Figure 1.

### Bioinformatics analyses

Survival analyses based on the mRNA expressions were conducted by Kaplan-Meier plotter (https://kmplot.com/analysis/)^30^ utilizing RNA-seq data and survival information from Gene Expression Omnibus (GEO; https://www.ncbi.nlm.nih.gov/geo/), European Genome-phenome Archive (EGA; https://egaarchive.org/), and The Cancer Genome Atlas (TCGA; https://www.cancer.gov/ccg/research/genomesequencing/tcga) with the best performing cutoff percentiles. Survival subset analyses according to histotypes were conducted by cBioPortal (https://www.cbioportal.org/) based on mRNA expression z-scores relative to all samples (log RNA Seq V2 RSEM) from TCGA, PanCancer Atlas with the median cutoff values.^31^ The correlation of gene expression with immune infiltration level was analyzed by TIMER2.0 (http://timer.comp-genomics.org/)^32^ utilizing quanTIseq algorithm.^33^ Rho and *P*-values were calculated by the Spearman’s rank correlations.

### Statistical analyses

Differences in proportions were evaluated by the Fisher’s exact test. Differences in continuous variables were evaluated by the Mann-Whitney *U* test or the Kruskal-Wallis test. The optimal cutoff values of IHC scores for the relationships with patient OS were calculated using the maximally selected log-rank statistic (Table S1—see online supplementary material). Kaplan-Meier survival curves were generated and compared by the log-rank test. The univariate and multivariate analyses for prognostic factors were conducted by the Cox proportional hazard model. R version 4.2.2 (https://www.r-project.org/) was used for all statistical analyses, and *P*-values < .05 were considered as statistically significant.

## Results

We evaluated the IHC expressions of TF and the related proteins as well as CD8 in 229 endometrial carcinomas (Table S1—see online supplementary material), and first examined the correlations among the protein expressions (Table S2—see online supplementary material). High expression of membrane TF (mTF) was associated with high expressions of cytoplasmic TF and VEGF (*P* = 2.2E-16 and .045; Table S2—see online supplementary material). High expression of cytoplasmic p-TF was associated with low expression of nuclear p-TF and high expression of VEGF (*P* = .00033 and .022; Table S2—see online supplementary material). High expression of PAR-1 was associated with high expression of PAR-2 (*P* = .016; Table S2—see online supplementary material).

Next, we examined the correlations between the protein expressions and the clinicopathologic factors (Table 2). High expression of mTF was associated with presence of VTE at the time of primary treatment or recurrence (*P* = .018; Table 2). High expressions of cytoplasmic TF and PAR-1 and low expression of cytoplasmic p-TF were all associated with aggressive histology (*P* = .042, 4.4E-12, and .029; Table 2). High expression of PAR-1 was associated with the presence of LVSI, while high expression of PAR-2 was associated with the absence of LVSI and superficial myometrial invasion (*P* = .03, .027, and .029). High expression of cytoplasmic p-TF was associated with younger age and higher body mass index (BMI), whereas high expression of PAR-1 was associated with lower BMI (*P* = .0068, .001, and .034; Table 2).

**Table 2:** Relationships between the protein expressions and clinicopathologic factors.

	TF (Membrane)			TF (Cytoplasm)			p-TF (Cytoplasm)			p-TF (Nuclear)			PAR-1			PAR-2			VEGF			CD8		
	High	Low		High	Low		High	Low		High	Low		High	Low		High	Low		High	Low		High	Low	
	(*n* = 40)	(*n* = 189)	*P*-value	(*n* = 28)	(*n* = 201)	*P*-value	(*n* = 139)	(*n* = 90)	*P*-value	(*n* = 188)	(*n* = 41)	*P*-value	(*n* = 121)	(*n* = 108)	*P*-value	(*n* = 96)	(*n* = 133)	*P*-value	(*n* = 77)	(*n* = 152)	*P*-value	(*n* = 206)	(*n* = 23)	*P*-value
**Age (≥60 vs. <60)**	26 (65%)	93 (49%)	0.082	15 (54%)	104 (52%)	1	62 (45%)	57 (63%)	0.0068	102 (54%)	17 (41%)	0.17	70 (58%)	49 (45%)	0.065	48 (50%)	71 (53%)	0.69	42 (55%)	77 (51%)	0.67	104 (50%)	15 (65%)	0.19
**BMI (≥30 vs. <30)**	6 (15%)	32 (17%)	1	4 (14%)	34 (17%)	1	32 (23%)	6 (7%)	0.0010	28 (15%)	10 (24%)	0.16	14 (12%)	24 (22%)	0.034	17 (18%)	21 (16%)	0.72	12 (16%)	26 (17%)	0.85	32 (16%)	6 (26%)	0.23
**Aggressive histology (vs. Endometrioid G1)**	28 (70%)	101 (53%)	0.078	21 (75%)	108 (54%)	0.042	70 (50%)	59 (66%)	0.029	110 (59%)	19 (46%)	0.17	94 (78%)	35 (32%)	4.4E-12	54 (56%)	75 (56%)	1	39 (51%)	90 (59%)	0.26	113 (55%)	16 (70%)	0.19
**Advanced stage (III/IV vs. I/II)**	8 (20%)	44 (23%)	0.84	8 (20%)	44 (23%)	0.47	36 (26%)	16 (18%)	0.20	42 (22%)	10 (24%)	0.84	29 (24%)	23 (21%)	0.64	19 (20%)	33 (25%)	0.43	20 (26%)	32 (21%)	0.41	49 (24%)	3 (13%)	0.30
**Deep myometrial invasion (≥1/2 vs. <1/2)**	15 (38%)	76 (40%)	0.86	11 (39%)	80 (40%)	1	52 (37%)	39 (43%)	0.41	75 (40%)	16 (39%)	1	53 (44%)	38 (35%)	0.22	30 (31%)	61 (46%)	0.029	28 (36%)	63 (41%)	0.48	82 (40%)	9 (39%)	1
**LVSI (present vs. absent)**	14 (35%)	73 (39%)	0.73	10 (36%)	77 (38%)	0.84	48 (35%)	39 (43%)	0.21	74 (39%)	13 (32%)	0.38	54 (45%)	33 (30%)	0.030	28 (29%)	59 (44%)	0.027	28 (36%)	59 (39%)	0.77	81 (39%)	6 (26%)	1
**Lymph node metastasis (present vs. absent)**	4 (10%)	22 (12%)	1	4 (14%)	22 (11%)	0.54	15 (11%)	11 (12%)	0.83	23 (12%)	3 (7%)	0.59	14 (12%)	12 (11%)	1	9 (7%)	17 (13%)	0.53	10 (13%)	16 (11%)	0.66	23 (11%)	3 (13%)	0.73
**VTE at the time of primary treatment or recurrence**	12 (30%)	26 (14%)	0.018	8 (29%)	30 (15%)	0.099	21 (15%)	17 (19%)	0.47	34 (18%)	4 (10%)	0.25	24 (20%)	14 (13%)	0.21	16 (17%)	22 (17%)	1	16 (21%)	22 (14%)	0.26	31 (15%)	7 (30%)	0.075

Abbreviations: BMI, body mass index; LVSI, lymphovascular space invasion; p-TF, phospho-tissue factor; TF, tissue factor; VTE, venous thromboembolism.

**Figure 2:**
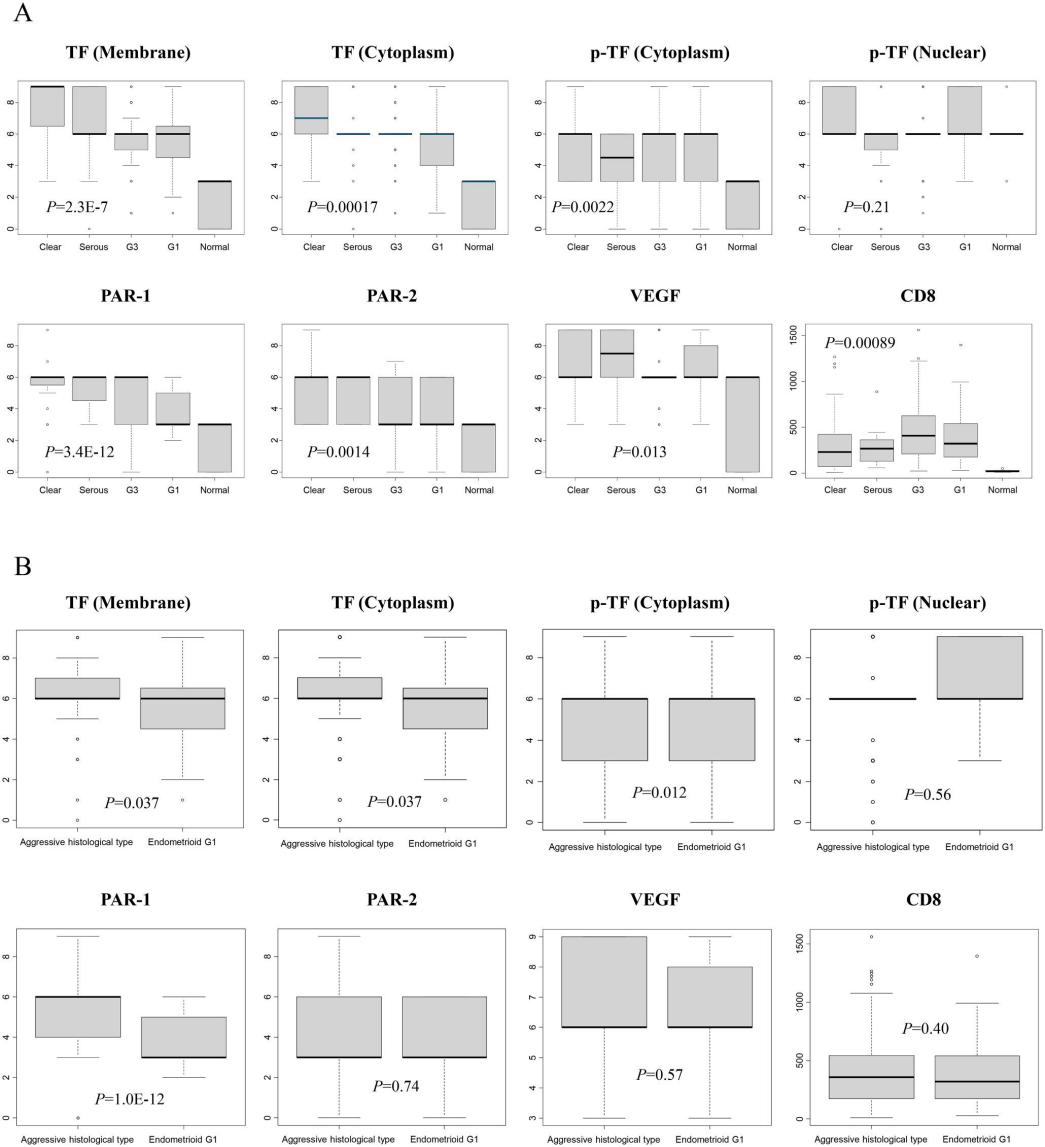
The expression levels of TF, the related proteins and CD8 in endometrial carcinomas. (A) Comparison of the protein expressions or the positive TIC count among histotypes. *P*-values were calculated by the Kruskal-Wallis test. (B) Comparison of the protein expressions or the positive TIC count between the aggressive histotypes and endometrioid carcinoma, G1. *P*-values were calculated by the Mann-Whitney *U* test.

We next examined the differences of the protein expression levels according to the different histotypes (Figure 2). There appeared trends that the expressions of membrane and cytoplasmic TF and PAR-1 were the highest in clear cell histology, followed by serous and endometrioid G3, and the lowest in endometrioid G1 histology, although not statistically significant (Figure 2A). The comparison of those expressions between the aggressive histology and endometrioid G1 showed statistical difference (*P* = .037, .037, and 1.0E-12; Figure 2B).

**Figure 3:**
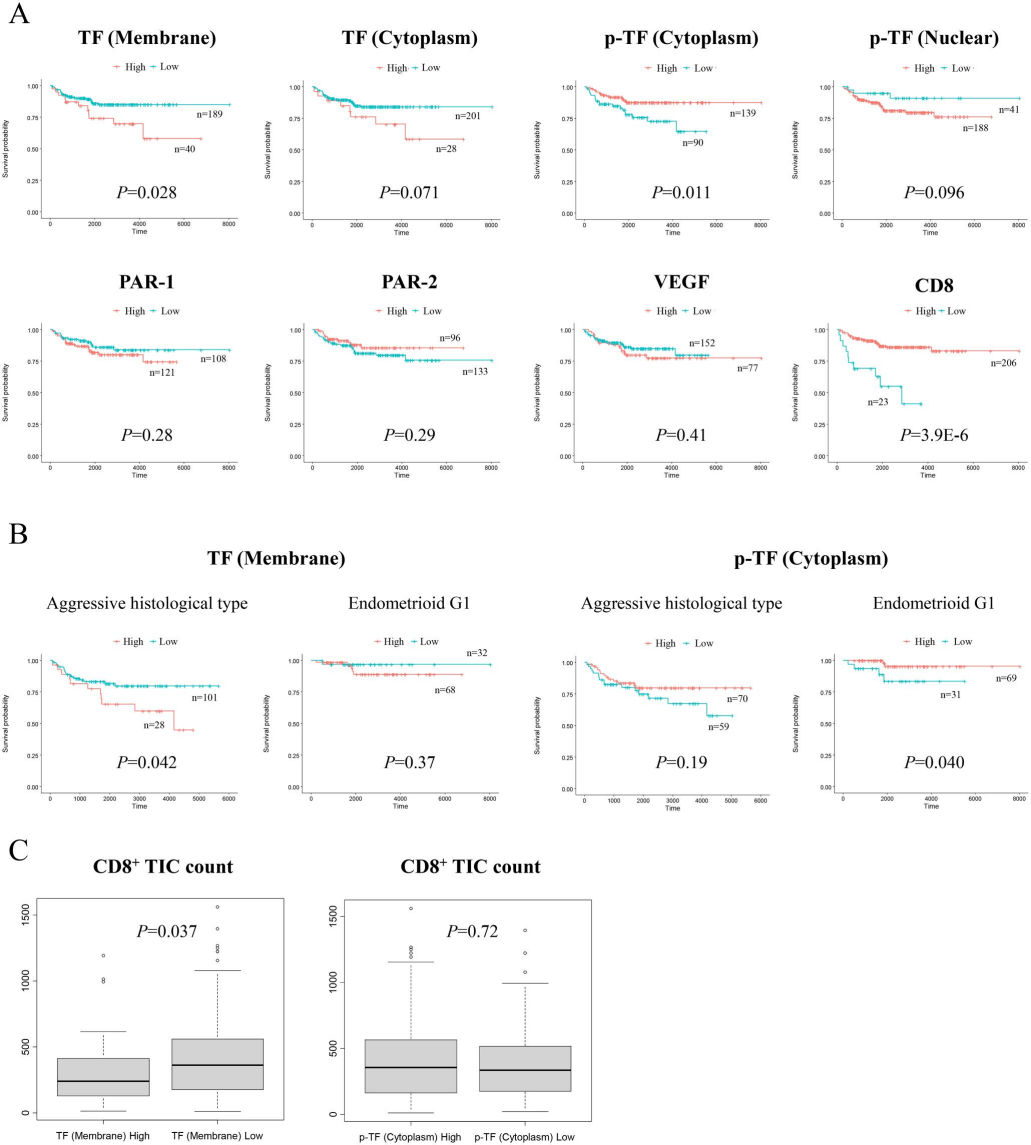
Comparison of survival curves or TIC count according to the protein expression levels or TIC count (high vs. low based on the cutoff values in Table S1—see online supplementary material). (A) OS curves in the whole group of patients according to the expressions or the CD8^+^ TIC count. *P*-values were calculated by the log-rank test. (B) OS curves in the patients with the aggressive histotypes or endometrioid carcinoma, G1 according to the expressions or the CD8^+^ TIC count. *P*-values were calculated by the log-rank test. (C) Comparison of the CD8^+^ TIC count between the tumors with high vs. low expressions of mTF or cytoplasmic p-TF. *P*-values were calculated by the Mann-Whitney *U* test.

We subsequently explored the prognostic roles of the proteins by comparing patient OS according to the expressions. High expression of mTF correlated with worse OS, whereas high expression of cytoplasmic p-TF and high density of CD8^+^ TICs both correlated with better OS (*P* = .028, .011, and .000039; Figure 3A). The expression of PAR-1, PAR-2 or VEGF exhibited no significant association with OS (Figure 3A). When we further looked into subset analyses by separating the patients by histologic types, mTF expression was associated with worse OS only in aggressive histotype, and cytoplasmic p-TF expression was associated with better OS only in endometrioid G1 (*P* = .042 and .040; Figure 3B). Next, the univariate analysis including the conventional prognostic factors for endometrial cancer and the protein expressions indicated that mTF, stage, muscular invasion, LVSI and histotype were significant for unfavorable OS, and that cytoplasmic p-TF was significant for favorable OS (*P* = .032, 5.2E-07, .0036, 1.6E-05, .0015, and .013; Table 3). The subsequent multivariate analysis with the significant factors from the univariate analysis revealed that mTF, stage and LVSI were significant and independent for unfavorable OS (*P* = .0087, .00040, and .014; Table 3), and that cytoplasmic p-TF was significant and independent for favorable OS (*P* = .012; Table 3). We further conducted those univariate and multivariate analyses separately in different histotypes (Table 3). Membrane TF was found to be significant and independent for unfavorable OS only in the aggressive histologic subtype (*P* = .0064; Table 3), while cytoplasmic p-TF was not found to be significant in the univariate analysis for either the aggressive or endometrioid G1 histology (Table 3). In order to further explore the underlying mechanism for the prognostic role of TF, we examined the correlations between CD8^+^ TIC count and the expression of mTF or cytoplasmic p-TF. Membrane TF expression level was found to be inversely associated with CD8^+^ TIC count, while cytoplasmic p-TF showed no such correlation (*P* = .037 and .72; Figure 3C).

**Table 3:** Univariate and multivariate analyses of the prognostic factors associated with OS in the whole group and in the histotypes.

	All histotypes (*n* = 229)						Aggressive histology (*n* = 129)						Endometrioid G1 (*n* = 100)					
	Univariate			Multivariate			Univariate			Multivariate			Univariate			Multivariate		
	HR	95% CI	*P*-value	HR	95% CI	*P*-value	HR	95%CI	*P*-value	HR	95%CI	*P*-value	HR	95%CI	*P*-value	HR	95%CI	*P*-value
**TF (Membrane)**	2.19	1.07-4.47	0.032	2.69	1.29-5.65	0.0087	2.14	1.01-4.54	0.047	2.9	1.35-6.24	0.0064	2.60	0.30-22.01	0.39	–	–	–
**TF (Cytoplasm)**	2.04	0.93-4.50	0.077	–	–	–	1.89	0.83-4.27	0.13	–	–	–	2.63	0.31-22.56	0.38	–	–	–
**p-TF (Cytoplasm)**	0.43	0.22-0.84	0.013	0.42	0.21-0.83	0.012	0.62	0.30-1.29	0.20	–	–	–	0.20	0.04-1.10	0.064	–	–	–
**p-TF (Nuclear)**	2.63	0.81-8.61	0.11	–	–	–	1.62	0.49-5.36	0.43	–	–	–	3.0.E+08	0-Inf	1	–	–	–
**PAR-1**	1.44	0.73-2.84	0.29	–	–	–	0.54	0.26-1.13	0.10	–	–	–	0.63	0.07-5.42	0.68	–	–	–
**PAR-2**	0.68	0.33-1.39	0.29	–	–	–	0.73	0.34-1.58	0.43	–	–	–	0.34	0.04-2.96	0.33	–	–	–
**VEGF**	1.32	0.68-2.59	0.41	–	–	–	1.29	0.61-2.74	0.50	–	–	–	2.71	0.55-13.49	0.22	–	–	–
**Age (≥60 vs. <60)**	1.44	0.73-2.85	0.29	–	–	–	1.51	0.70-3.26	0.29	–	–	–	0.63	0.12-3.45	0.60	–	–	–
**Stage (I-II vs. III-IV)**	5.58	2.85-10.92	5.2E-07	3.76	1.81-7.82	0.00040	3.81	1.83-7.94	0.00036	3.08	1.36-6.97	0.0070	12.28	2.24-67.20	0.0038	6.07	1.68-34.53	0.042
**MI (≥1/2 vs. <1/2)**	2.73	1.39-5.38	0.0036	1.25	0.59-2.68	0.56	2.21	1.06-4.60	0.035	1.12	0.47-2.63	0.80	9.43	1.10-80.72	0.041	2.70	0.28-25.97	0.39
**LVSI (present vs. absent)**	5.04	2.42-10.51	1.6E-05	2.90	1.25-6.77	0.014	2.95	1.34-6.49	0.0072	2.26	0.90-5.68	0.083	19.26	2.24-165.28	0.0070	8.74	0.90-84.88	0.062
**Histotype (aggressive vs. G1)**	4.15	1.72-9.99	0.0015	2.11	0.84-5.29	0.11	–	–	–	–	–	–	–	–	–	–	–	–

Abbreviations: CI, confidence interval; HR, hazard ratio; LVSI, lymphocascular space invasion; MI, myometrial invasion; OS, overall survival; p-TF, phospho-tissue factor; TF, tissue factor.

**Figure 4:**
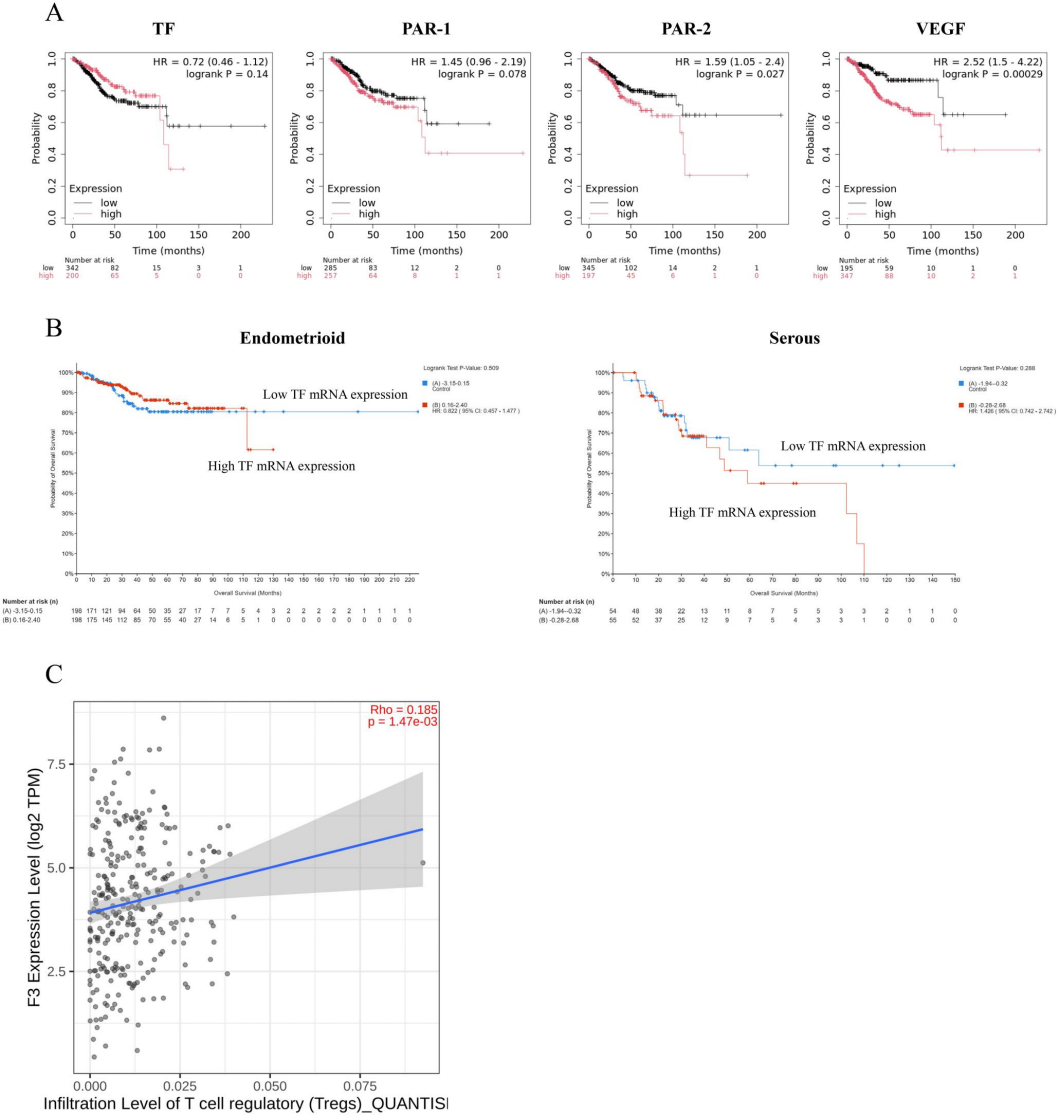
Bioinformatics survival and immune infiltration analyses in endometrial cancer. (A) OS curves based on the mRNA expressions by Kaplan-Meier plotter. *P*-values were calculated by the log-rank test. (B) OS curves based on the TF mRNA expression in the subset of endometrioid carcinomas or serous carcinomas by cBioPortal. *P*-values were calculated by the log-rank test. (C) Correlation of TF expression with Treg-cell infiltration in endometrial cancer by TIMER2.0. Rho and *P*-values were calculated by the Spearman’s rank correlations.

We further conducted bioinformatics analyses utilizing the TCGA data (Figure 4). By Kaplan-Meier plotter, high mRNA expressions of TF and PAR-1 exhibited trends toward unfavorable OS, and high mRNA expressions of PAR-2 and VEGF exhibited association with unfavorable OS (*P* = .14, .078, .027, and .00029; Figure 4A). We also conducted subset analysis separating patients based on histotypes by cBioPortal. For endometrioid histology, patients with high and low TF mRNA expressions showed similar OS, while those with high expression appear to have worse OS than those with low expression, although no statistical difference was observed (hazard ratio [HR]: 0.82 *vs.* 1.4; *P* = .51 *vs.* .29; Figure 4B).

## Discussion

Our survival analysis exhibited that high expression of mTF correlated with worse OS, while high expression of cytoplasmic p-TF correlated with better OS (Figure 3A), indicating that mTF and cytoplasmic p-TF are playing the opposite prognostic roles. The analysis of the associations between protein expressions and clinicopathologic factors showed that high expression of mTF was associated with presence of VTE at the time of primary treatment or recurrence, while high expression of p-TF was associated with non-aggressive histotype (Table 2). In the tumor microenvironment, activation of platelets are known to trigger the TF-mediated extrinsic coagulation pathway leading to tumor-associated thrombogenesis.^4^ Activated platelets also contribute to tumor growth, angiogenesis and metastasis as well as immune evasion through secreting multiple cytokines.^34–36^ TF expression reportedly causes malignant potential *in vitro* and *in vivo.*^37–39^ On the contrary, phosphorylation of Ser258 in the cytoplasmic domain of TF, which can be detected by the anti-p-TF antibody that we used in the present study, reportedly suppresses TF release into microvesicles (MVs), carriers for TF procoagulant activity.^5^ In vascular endothelial cells, TF release into MVs was induced by aspartate substitution (to mimic phosphorylation) of Ser253, reduced by that of Ser258, and prolonged by alanine substitution (to prevent phosphorylation) of Ser258 within the TF cytoplasmic domain.^40^ Furthermore, knocking-down filamin-A, an actin-binding cytoskeletal protein regulating the function and cellular localization of numerous proteins,^41^ inhibited PAR-2-induced MV incorporation and procoagulant activity of TF.^42^^,^^43^  *In vitro* binding assays indicated the opposing roles of Ser253 and Ser258 phosphorylation in the interaction between TF and filamin-A, with the highest affinity for phospho-Ser253 and the lowest for phospho-Ser258.^42^ Therefore, it should be reasonable that mTF was found to correlate with VTE and unfavorable prognosis, and that cytoplasmic p-TF was found to correlate with non-aggressive histotype and favorable prognosis. Additionally, mTF expression level was observed to inversely correlate with CD8^+^ TIC count (Figure 3C). Besides, the TCGA data analysis by TIMER2.0 indicated that TF expression level positively correlated with Treg-cell infiltration (Figure 4C). CD8^+^ cytotoxic T cells are known to be suppressed by Treg cells via multiple pathways including secretion of cytokines TGF-β, IL-10 and IL-35, binding of IL-2 to IL-2Rα and secretion of perforins and granzymes.^44^ Accordingly, it can be assumed that mTF inhibits CD8^+^ cells through upregulating Treg cells, resulting in poor prognosis (Figure S1—see online supplementary material for a color version of this figure). Indeed, TF is reported to be involved in immune evasion in other kinds of tumor cells.^45–47^ Further experiments such as exploring TF’s function on Treg-cell infiltration are required to clarify the prognostic roles and their mechanisms by TF in endometrial carcinoma. Additionally, whether TF expression fluctuates during treatment, progression, or recurrence is another important question for prognostic utility, needing future investigation.

In the following subset analyses of separating patients into histotypes, the prognostic significances were observed only in aggressive histotypes for mTF or endometrioid G1 histology for cytoplasmic p-TF (Figure 3B), implicating that the prognostic roles of TF may be histotype specific. Differential prognostic roles of TF according to histotypes were also observed and supported by the bioinformatics analyses using the TCGA data, where the prognostic effect of TF appeared different between endometrioid and serous carcinomas (Figure 4B). Our univariate and multivariate analyses found mTF and cytoplasmic p-TF to be the opposite and independent prognostic factors (Table 3), suggesting that inhibiting mTF and/or dephosphorylating cytoplasmic TF may improve patients’ survival. The subsequent subset analyses showed that mTF was a significant and independent unfavorable prognostic factor even in the aggressive histotype alone with the HR equivalent to stage (Table 3), demonstrating the potential usefulness of mTF as a biomarker for the therapeutic target to improve the prognosis of this refractory cohort of patients.

In a phase 3 trial, innovaTV 301, which evaluated TV monotherapy or chemotherapy as second-line or third-line therapy in 502 patients with recurrent or metastatic cervical cancer, the median OS (11.5 vs. 9.5 months; HR, 0.70; 95% CI, 0.54-0.89; *P* = .004) and progression-free survival (4.2 vs. 2.9 months; HR, 0.67; 95% CI, 0.54-0.82; *P* < .001) were shown to be significantly longer in the TV group than in the chemotherapy group. The confirmed objective response rate was 17.8% vs. 5.2% (odds ratio, 4.0; 95% CI, 2.1-7.6; *P* < .001).^26^ Based on these results, TV was recently approved by the FDA for the treatment of advanced or recurrent cervical cancer. As regards endometrial cancer, a phase 1-2 study, innovaTV 201, evaluated TV in patients with advanced or metastatic solid tumors including 14 endometrial cancers in the dose-expansion phase.^48^ The objective response rates were 26.5% (9/34) for cervical cancer and 7.1% (1/14) for endometrial cancer. Regarding the positivity rate of TF expression in tumors, TF was reported to be highly expressed in cervical cancer, 100% of tumor tissues (8/8) by IHC.^17^ As for endometrial cancer, TF expression was observed in 100% (16/16) of serous carcinomas by IHC, but not in normal endometrium.^20^ In another study, TF expression was present in 41.17% (7/17) of clear cell carcinomas, 13.3% (2/15) of G1 endometrioid carcinomas, and 10% (2/20) of serous carcinomas.^19^ In our study, membrane and cytoplasmic TF expressions were both the highest in clear cell carcinoma, followed by serous carcinoma, endometrioid G3 and G1, and the lowest in normal endometrium (Figure 2A). Those expressions were both significantly higher in the aggressive histotype than in endometrioid G1 (Figure 2B). Although the detailed information about histotypes in the innovaTV 201 study is unknown,^48^ most of endometrial cancers are usually endometrioid G1. Therefore, we infer that the efficacy of TV will increase when cases of the aggressive histotypes are selected for the treatment subjects. Clinical studies evaluating the efficacy of TV in endometrial cancers of the aggressive histotypes are warranted.

In our analysis of the relationships between the TF-related protein expressions and clinicopathologic factors indicated that cytoplasmic p-TF was significantly associated with younger age, higher BMI and endometrioid G1 histology (Table 2). By the conventional classification of endometrial cancer,^49^ all of these factors are included in the characteristics of type I tumor with favorable prognosis, and our survival analysis also exhibited favorable prognostic effect of cytoplasmic p-TF (Figure 3A). In a hyperglycemic or diabetic setting, the elevated levels of diacylglycerol reportedly resulted in enhanced activation and synthesis of protein kinase C (PKC).^50^ PKC in turn induced Ser258 phosphorylation of cytoplasmic domain of TF in vascular endothelial cells.^51^^,^^52^ High BMI is well known to be associated with insulin resistance, which is considered to be involved in type I tumorigenesis. Hence, it is reasonable that cytoplasmic p-TF correlates with the characteristics of type I tumor, while further experiments such as investigating the effect of high glucose on the PKC-p-TF signaling are necessary to validate this hypothesis. The TCGA molecular classification, which is more informative for predicting prognosis than the conventional classification, was recently integrated into the FIGO staging system.^53^ It is not possible, however, to correlate the protein expressions in the present study with this molecular profile, as the genetic testing was conducted for not all our samples. Although we examined the association between the TF and TP53 mRNA expressions by the bioinformatics analysis in endometrial cancer, no significant relationship was observed (data not shown).

As therapeutics other than ADCs, a couple of antibodies against TF have been investigated for cancer treatment in preclinical settings.^54^^,^^55^ However, increased risk of hemorrhage is concerned for inhibiting the TF signaling. Another possible approach may be phosphorylating the cytoplasmic domain of TF, which is supposed to suppress the TF activity,^40^^,^^42^ based on our univariate and multivariate analysis (Table 3). Our findings also showed that cytoplasmic p-TF expression did not correlate with VTE by contrast with mTF expression (Table 2), suggesting the mediation of different signaling pathways. Combining the both therapeutic approaches may be another option for decreasing the risk of severe adverse toxicities.

There are several limitations in this study. First, this is a retrospective study that contains possible selection biases. Second, the sample size in the subgroup analyses is relatively small, needing cautious interpretation. Third, our results are based mainly on semiquantitative analyses by IHC, requiring validation by quantitative image or mRNA analyses. Yet we also performed bioinformatics analyses by utilizing the mRNA quantitative data of TCGA, some of which resemble and support our results. Functional assays are necessary to mechanistically validate the TF’s prognostic role. Lastly, the cases with the aggressive histology were included consecutively, while the cases with endometrioid G1 histology were randomly selected from the study period (Figure S2—see online supplementary material for a color version of this figure). Therefore, the prevalence of each histotype in the whole study cohort does not reflect that of the real population, affecting the prognostic significances in the survival analyses. However, the statistical significances of mTF and cytoplasmic p-TF remained even in the subset analyses on the aggressive or endometrioid G1 histology, except for cytoplasmic p-TF in the univariate and multivariate analysis (Figure 3B and Table 3). Despite these limitations, the strength of the present study is that, to our knowledge, this is the first report investigating the prognostic and clinicopathologic significances of TF in endometrial cancer.

## Conclusion

Our expression profile and bioinformatics analyses presented here revealed the histology-specific prognostic roles of TF, presenting the potential usefulness as a biomarker for the therapeutic target to improve the prognosis of patients with aggressive diseases, although further mechanistic investigations particularly on the TF’s immunologic and metabolic functions and their phosphoserine residues’ involvement is required (Figure S1—see online supplementary material for a color version of this figure). The current findings propose that TF-directed drugs such as TV can be incorporated into novel strategies for refractory patients. Future clinical trials assessing the efficacy of TV in endometrial carcinoma specifically of aggressive histotype are warranted.

## Supplementary Material

oyag053_Supplementary_Data

## Data Availability

The data used and/or analyzed in this study are available from the corresponding author on reasonable request.
