# Supplementary material for: Prognostic and histologic significances of the expression profile of membrane tissue factor for aggressive endometrial carcinomas
Source: Oncologist. 2026 Feb 23;31(4):oyag053. doi: 10.1093/oncolo/oyag053 (PMC13006067; doi:10.1093/oncolo/oyag053)
Supplement: oyag053_Supplementary_Data [file oyag053_supplementary_data.zip › Supplementary Figure Captions_revised.docx]

**Supplementary Figure 1.** Proposed pathways of TF-mediated tumor biology and the summary of our results and hypothesis. TF expressed on tumor cells binds to and activates FVII, leading to formation of FXa-FVa complex on activated platelets. Activation of thrombin follows, resulting in thrombogenesis. Activated thrombin in turn activates PAR-1, which inhibits CD8a^+^ T cells infiltration, leading to immune evasion. TF-FVIIa complex activates PAR-2, leading to tumor growth. PAR-2 phosphorylates Ser253 within the cytoplasmic domain of TF, inducing TF release into MVs, which is inhibited by phosphorylation of Ser258 of the cytoplasmic domain. TF-TVIIa-PAR-2 complex and activated platelets promote VEGF production, inducing angiogenesis, migration, metastasis and dissemination. Our IHC results in endometrial carcinomas showed that mTF expression correlated with aggressive histology, VTE, low CD8^+^ TIC count and worse OS, whereas pSer258-cytoplasmic TF (cTF) expression correlated with endometrioid G1 histology, higher BMI, younger age and better OS. Bioinformatics analysis showed that TF expression positively correlated with Treg infiltration, suggesting that the detrimental prognostic role of mTF involves immune evasion via promoting Treg infiltration. Created in BioRender. Minaguchi, T. (2026) https://BioRender.com/8oi2isn.

**Supplementary Figure 2.** Flow chart of patient selection.
