# Supplementary material for: Prognostic and histologic significances of the expression profile of membrane tissue factor for aggressive endometrial carcinomas
Source: Oncologist. 2026 Feb 23;31(4):oyag053. doi: 10.1093/oncolo/oyag053 (PMC13006067; doi:10.1093/oncolo/oyag053)
Supplement: oyag053_Supplementary_Data [file oyag053_supplementary_data.zip › Updated_Supplementary_Data.zip!/Updated_Supplementary_Data/Oncol_TF_Supplementary Figure 1_revised.pdf]

Figure S1

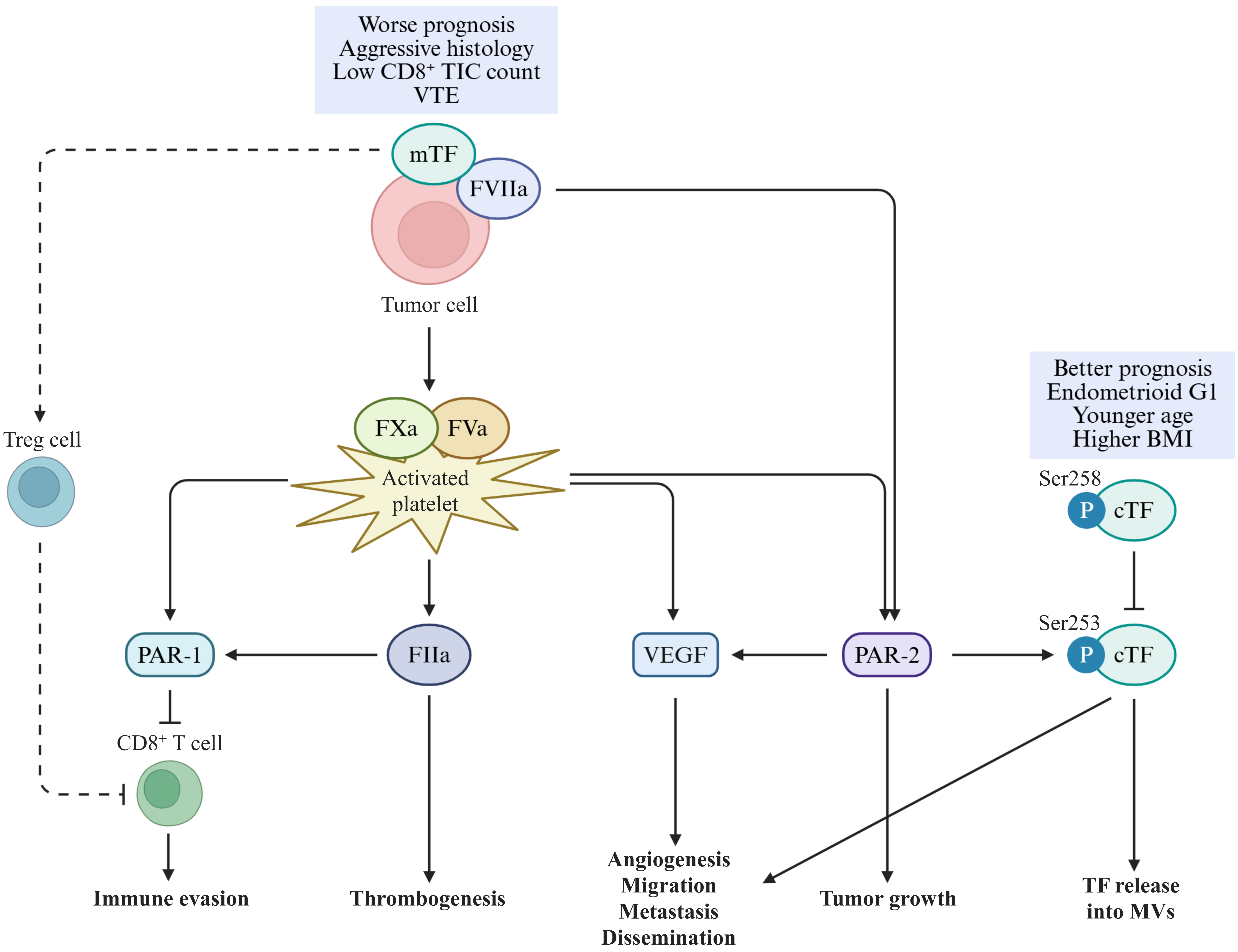
Worse prognosis
Aggressive histology
Low CD8+ TIC count
VTE
mTF
FVIIa
Tumor cell
Treg cell
FXa
FVa
Activated platelet
Better prognosis
Endometrioid G1
Younger age
Higher BMI
Ser258
P
cTF
Ser253
P
cTF
PAR-1
FIIa
VEGF
PAR-2
CD8+ T cell
Immune evasion
Thrombogenesis
Angiogenesis
Migration
Metastasis
Dissemination
Tumor growth
TF release
into MVs
