# Supplementary material for: Prognostic and histologic significances of the expression profile of membrane tissue factor for aggressive endometrial carcinomas
Source: Oncologist. 2026 Feb 23;31(4):oyag053. doi: 10.1093/oncolo/oyag053 (PMC13006067; doi:10.1093/oncolo/oyag053)
Supplement: oyag053_Supplementary_Data [file oyag053_supplementary_data.zip › Updated_Supplementary_Data.zip!/Updated_Supplementary_Data/Oncol_TF_Supplementary Figure 2.pdf]

Figure S2

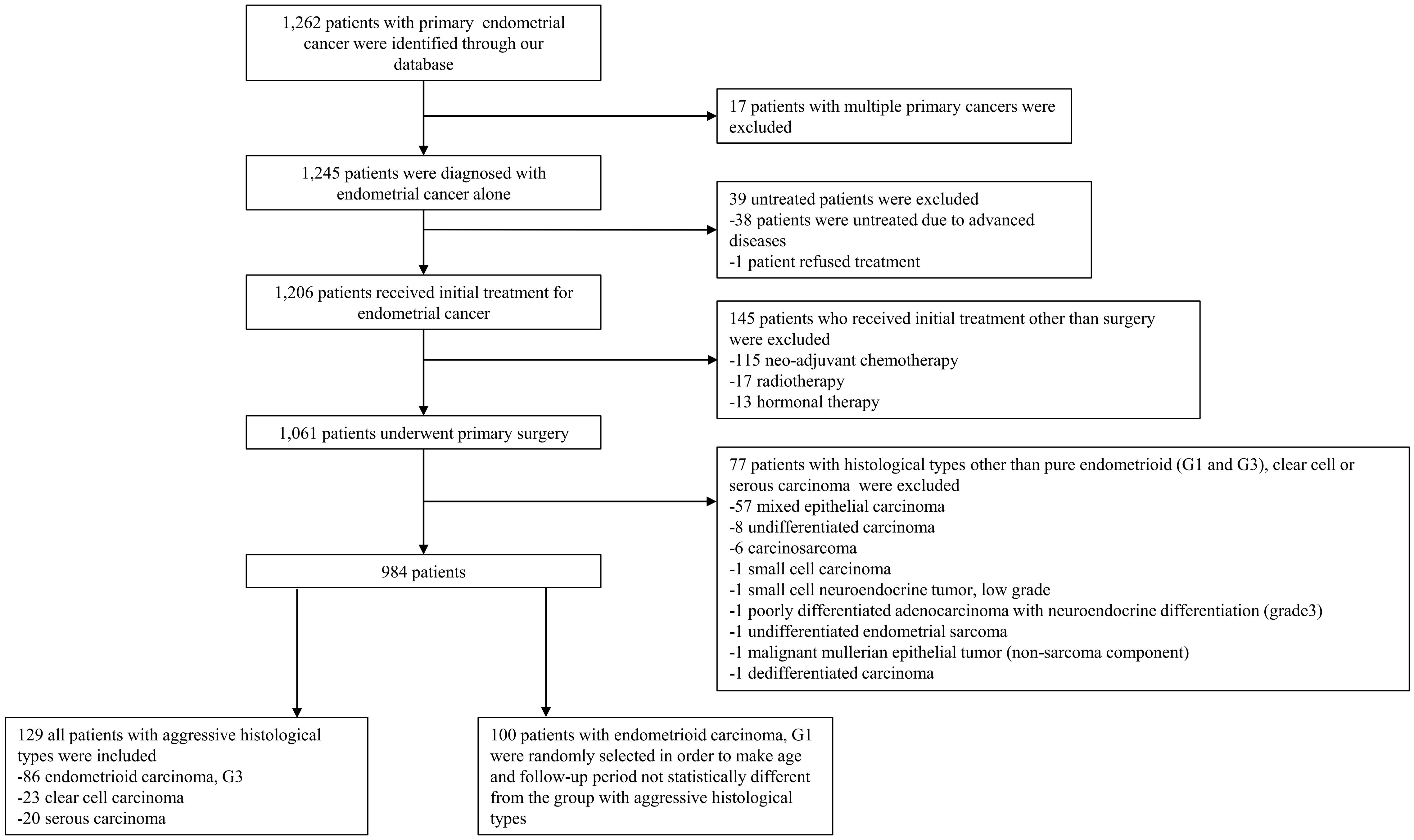

1,262 patients with primary endometrial cancer were identified through our database
17 patients with multiple primary cancers were excluded
1,245 patients were diagnosed with endometrial cancer alone
39 untreated patients were excluded
-38 patients were untreated due to advanced diseases
-1 patient refused treatment
1,206 patients received initial treatment for endometrial cancer
145 patients who received initial treatment other than surgery were excluded
-115 neo-adjuvant chemotherapy
-17 radiotherapy
-13 hormonal therapy
1,061 patients underwent primary surgery
77 patients with histological types other than pure endometrioid (G1 and G3), clear cell or serous carcinoma were excluded
-57 mixed epithelial carcinoma
-8 undifferentiated carcinoma
-6 carcinosarcoma
-1 small cell carcinoma
-1 small cell neuroendocrine tumor, low grade
-1 poorly differentiated adenocarcinoma with neuroendocrine differentiation (grade3)
-1 undifferentiated endometrial sarcoma
-1 malignant mullerian epithelial tumor (non-sarcoma component)
-1 dedifferentiated carcinoma
984 patients
129 all patients with aggressive histological types were included
-86 endometrioid carcinoma, G3
-23 clear cell carcinoma
-20 serous carcinoma
100 patients with endometrioid carcinoma, G1 were randomly selected in order to make age and follow-up period not statistically different from the group with aggressive histological types
