# Supplementary material for: Prognostic and histologic significances of the expression profile of membrane tissue factor for aggressive endometrial carcinomas
Source: Oncologist. 2026 Feb 23;31(4):oyag053. doi: 10.1093/oncolo/oyag053 (PMC13006067; doi:10.1093/oncolo/oyag053)
Supplement: oyag053_Supplementary_Data [file oyag053_supplementary_data.zip › Updated_Supplementary_Data.zip!/Updated_Supplementary_Data/Oncol_TF_Supplementary Table 1.pdf]

Table S1. Optimal cut-off values of IHC scores for the relationship with OS.

	Mean±SD	Min	Max	Cut-off	Category	N (%)
TF (Membrane)	6.0±1.8	0	9	7<	High	40 (17%)
					Low	189 (83%)
TF (Cytoplasm)	5.8±1.7	0	9	7<	High	28 (12%)
					Low	201 (88%)
p-TF (Cytoplasm)	5.1±2.0	0	9	3<	High	139 (61%)
					Low	90 (39%)
p-TF (Nuclear)	6.1±2.1	0	9	3<	High	188 (82%)
					Low	41 (18%)
PAR-1	4.6±1.5	0	9	4<	High	121 (53%)
					Low	108 (47%)
PAR-2	4.2±1.6	0	9	3<	High	96 (42%)
					Low	133 (58%)
VEGF	6.7±1.6	3	9	6<	High	77 (34%)
					Low	152 (66%)
CD8	393±295	11	1563	75<	High	206 (90%)
					Low	23 (10%)

Abbreviation: *IHC* immunohistochemistry, *OS* overall survival, *SD* standard deviation, *Min* minimum, *Max* maximum, *TF* tissue factor, *p-TF* phospho-tissue factor
