# Supplementary material for: Prognostic and histologic significances of the expression profile of membrane tissue factor for aggressive endometrial carcinomas
Source: Oncologist. 2026 Feb 23;31(4):oyag053. doi: 10.1093/oncolo/oyag053 (PMC13006067; doi:10.1093/oncolo/oyag053)
Supplement: oyag053_Supplementary_Data [file oyag053_supplementary_data.zip › Updated_Supplementary_Data.zip!/Updated_Supplementary_Data/Oncol_TF_Supplementary Table 2_revised.pdf]

Table S2. Relationships among the protein expressions.

	TF (Cytoplasm)			p-TF (Cytoplasm)			p-TF (Nuclear)			PAR-1			PAR-2			VEGF			CD8		
	High (n=28)	Low (n=201)	*P* -value	High (n=139)	Low (n=90)	*P* -value	High (n=188)	Low (n=41)	*P* -value	High (n=121)	Low (n=108)	*P* -value	High (n=96)	Low (n=133)	*P* -value	High (n=77)	Low (n=152)	*P* -value	High (n=206)	Low (n=23)	*P* -value
High TF expression (Membrane)	28 (100%)	12 (6%)	2.2E-16	26 (19%)	14 (16%)	0.60	33 (18%)	7 (17%)	1	26 (21%)	14 (13%)	0.12	20 (21%)	20 (15%)	0.29	19 (25%)	21 (14%)	0.045	33 (16%)	7 (30%)	0.14
High TF expression (Cytoplasm)	-	-	-	19 (14%)	9 (10%)	0.54	24 (13%)	4 (10%)	0.79	17 (14%)	11 (10%)	0.42	16 (17%)	12 (9%)	0.10	13 (17%)	15 (10%)	0.14	22 (11%)	6 (26%)	0.044
High p-TF expression (Cytoplasm)	-	-	-	-	-	-	104 (55%)	35 (85%)	0.00033	71 (59%)	68 (63%)	0.59	64 (67%)	75 (56%)	0.13	55 (71%)	84 (55%)	0.022	125 (61%)	14 (61%)	1
High p-TF expression (Nuclear)	-	-	-	-	-	-	-	-	-	106 (88%)	82 (76%)	0.025	76 (79%)	112 (84%)	0.38	64 (83%)	124 (82%)	0.86	169 (82%)	19 (83%)	1
High PAR-1 expression	-	-	-	-	-	-	-	-	-	-	-	-	60 (63%)	61 (46%)	0.016	43 (56%)	78 (51%)	0.58	105 (51%)	16 (70%)	0.12
High PAR-2 expression	-	-	-	-	-	-	-	-	-	-	-	-	-	-	-	32 (42%)	64 (42%)	1	84 (41%)	12 (52%)	0.37
High VEGF expression	-	-	-	-	-	-	-	-	-	-	-	-	-	-	-	-	-	-	67 (33%)	10 (43%)	0.35

Abbreviation: *TF* tissue factor, *p-TF* phospho-tissue factor
